# Helping themselves and helping others: how the passage of time influences why mothers with addictions take part in research

**DOI:** 10.3389/fpsyt.2023.1204882

**Published:** 2023-10-04

**Authors:** Karen Crawford, Lynda Russell, Sharon Graham, Fiona Turner

**Affiliations:** Mental Health and Wellbeing, School of Health and Wellbeing, University of Glasgow, Glasgow, United Kingdom

**Keywords:** mother, participation in research, doubly vulnerable, addiction, child welfare, altruism, empowerment, personal benefits

## Abstract

**Introduction:**

Women with addiction issues are under-researched, despite previous evidence that women’s needs are less understood than men’s and that services can overlook gender-specific issues. The majority of women in treatment are mothers and a significant number have contact with child welfare services. The voices of these women are needed to shape and influence evidence-based treatment and service development.

**Aim:**

To examine reasons and rationale for participation in research in mothers with addiction issues and involvement with the child welfare system.

**Method:**

Reflexive thematic analysis was used on interview transcripts from two qualitative studies. Individual themes from each study were combined and analysed to develop themes covering both studies and at different timepoints in process of child welfare assessment or removal of child/ren.

**Results:**

Three themes were identified (1) altruism; (2) personal benefit; and (3) empowerment. These mothers wanted to help with research. However, they also participated with the hope that this might facilitate the return of their children or help them to access support or services. A change over time was evident and, in those further down the line from child removal, there was a stronger want for their voices to be heard in order to advocate for other women and create change in services.

## Introduction

Men outnumber women in drug and alcohol treatment services, and this can result in women’s needs being overlooked (1–4). In Scotland, rates of drug related deaths are higher than the rest of the United Kingdom and Europe (5), with women’s deaths increasing at a faster rate than men (6). It is more important than ever to better understand the gender-specific needs of women with addictions to improve treatment, support services and outcomes for women.

Some gender-specific issues are already known, such as women having a poorer uptake of addiction services (7) and a greater likelihood of presenting with histories of trauma and interpersonal issues (1, 3, 4). Mothers with an addiction are also more likely than fathers to be primary carers for their children, impacting on the time and resources that allows their full engagement with services (8, 9). There are also concerns that women in addiction services are under researched (2, 10) making it unclear if services are offering evidence-based interventions.

High rates of women engaged with addiction treatment services are mothers and many of those are involved with child welfare services (10, 11). Women who experienced the removal of their children through child welfare proceedings describe feeling stigmatised and powerless (12). The lack of research into their needs may contribute to this experience and their participation in research can provide a much-needed insight into their lived experience.

Women with addictions who have had children removed can be viewed as ‘doubly vulnerable’ (13); vulnerable because of their addiction and any associated mental health issues, and vulnerable because of their child welfare circumstances. Care must be taken with conducting research with people who could be termed ‘vulnerable’ in order to attempt to uphold the ethical integrity of research (14). When ‘doubly vulnerable’ we tend to exclude these individuals from research (15), thus avoiding ethical dilemmas.

When recruiting and retaining participants in research, gatekeeping can be amplified where professionals are worried about potential harm because of a participant’s diminished competence, powerlessness, or disadvantaged status (16), jeopardising the generalisability of results (17). Gatekeepers “face an ethical conflict between enabling potential participants to exercise their right to choose whether or not to participate in the study and protecting potential volunteers against the perceived risk for undue harm” (18). When considering women with addictions, professionals may be worried about capacity to consent or that the timing of the participation is too risky in terms of the women’s recovery or wellbeing.

These common barriers and dilemmas prompted us to explore, from their own perspective, mothers’ reasons for taking part in two different studies involving mothers with addiction issues whose children had been removed from their care:

Study 1: a nested qualitative study exploring consent processes within the process evaluation of the Best Services Trial (BeST?), a Randomised Controlled Trial (RCT) which measures the effectiveness of an infant mental health approach when under 5 s are removed from the care of their parents. For more information on BeST? See Crawford et al. (19).

Study 2: a qualitative study focused on the experience of having children removed and contact with services in women within a Scottish Drug and Alcohol Recovery Service.

### Research aim and question

This brief research report aims to better understand why ‘doubly vulnerable’ mothers with addiction issues would take part in research, particularly as there is potential for the research to touch on the sensitive issues surrounding the removal of their children at a time where there may be multiple demands on their time from statutory services.

Research question: what are these mothers’ reasons and rationale for taking part in research?

## Methodology

### Design

This study is qualitative in design and is a secondary analysis of 30 semi-structured interviews with mothers in studies 1 and 2 above. Data were collected between 2012 and 2021, at which point the research questions were broader than that of the current study, and all data relevant to the current research question was extrapolated from the transcripts. Study 1 (Mothers in BeST?; MB) provided 18 transcripts and Study 2 (Mothers experience of removal; MR) provided 12 transcripts. Interviews lasted between 20 and 135 min (mean 73 min). Additional information about the studies can be found in Supplementary material.

### Data collection

In Study 1, interviews were conducted by FT and KC face-to-face in participant’s home or by telephone while in Study 2 they were conducted in an NHS Health Centre by LR. In the original studies, all data were collected by one-to-one semi-structured interviews, audio recorded and transcribed with consent. Transcripts were anonymised by removing any identifiable information (such as location, service names, and names of children, partners, family, and workers) and all mothers were allocated a pseudonym.

### Description of the sample

Participants in the pooled dataset included 30 birth mothers with addictions issues, contact with child welfare services and had/have child/ren removed from their care. Additional details for context are provided in Table 1.

**Table 1:** Demographic factors for sample.

Demographic factors	Years
Mean age	34 years
Age Range	21–49 years
Time since involvement with social work or removal of children	0 months–20 years
Child age at assessment or removal	Birth–7 years

### Ethics statements

Ethical approval was granted by West of Scotland NHS Research Ethics Service for both studies (Study 1—15/WS/0280; Study 2—17/WS/0255).

### Data analysis

We based our design on the framework developed by Haynes and colleagues (20), where multiple qualitative datasets were combined for analysis. Both studies had commonalities in participants, design and methodology, such as familiarity of the concept and data collection methods of the original studies. In addition, the original studies plus this analysis were approached from a similar epistemological position of phenomenology.

Data were analysed using Braun and Clarke’s six phases of reflexive thematic analysis (21) as this approach is suitable for a variety of theoretical frameworks and allows for flexibility in analytic scope. Due to the limited research in this area and our phenomenological stance, we used an inductive orientation to ensure that our analysis was driven by the data. To ensure adherence to rigour and fidelity to the analytical approach, we were guided at all stages by Braun and Clarke’s quality checklist (22) and their guidance on quality practice and reporting (23).

KC (Study 1) and LR (Study 2) reviewed data from their own studies. Individually, each went through every transcript and identified any lines or sections where participants discussed their motivations for participating in the respective studies. This data was then used to create new transcripts relating only to their reasons for participation in the study. Following Braun and Clarke’s guidelines, KC and LR began familiarisation to become immersed in the data. This led to semantic and latent coding of the data and the generation and revision of themes. An individual report on the themes developed was produced for each data set. Following this individual process, FT repeated the process with the transcripts and themes from the individual data sets and developed themes for the combined dataset. This time the analysis, while still taking an inductive orientation, focused on areas of similarity and difference to see if motivation and reasons for participating in research were consistent or changed over time and between groups. SG reviewed the themes for the combined dataset and gave feedback as an expert by experience.

### Researcher characteristics and reflexivity

The authors of this paper have various perspectives on the issues that this paper highlights: a clinical psychologist involved in research and clinical work with these mothers; a trial manager recruiting this target group; a mother with lived experience of addiction and child removal, who has supported women in similar situations and works in research; and a qualitative researcher and psychologist who has conducted substantial research with various stakeholders involved in providing support to this group. These multiple perspectives are viewed by the authors as an analytical strength in reflexive thematic analysis where subjectivity is viewed as a key feature (21). That said, we do recognise that our roles and experiences are likely to make us more inclined to support participation of ‘doubly vulnerable’ participant groups in research.

## Results

Three themes were identified for understanding participants’ reasons for participation in research, which are titled as altruism, personal benefits and empowerment. Altruism describes reasons for research participation in relation to helping others, personal benefits in relation to helping themselves, or improving their own situation and empowerment explores motivations for using research as a vehicle for using their lived experience to speak up and make change. See Table 2 for the themes and subthemes.

**Table 2:** Themes and subthemes.

Theme	Subtheme
Altruism	
Personal benefits	Participating to aid the return of their child/ren from care via the perceived mechanism of co-operation
	Participating to gain access to services and support
	Participating to help their future selves
Empowerment	Being heard
	Giving voice to others

As altruism has already been found to be a common factor for participating in research, we will describe this theme briefly and focus instead on the other two themes which provide a more nuanced insight into notions of “helping others” and other reasons for participating in research and highlight the temporal aspect of the themes depending on where mothers were in their timeline of assessment or removal of their children.

### Altruism

In common with participants in research generally, many of the mothers taking part in both studies felt that the research was an opportunity to use their situation to generally “help” with research “*I hope it helps*” (MR Lynne); with some mothers having an overarching goal of helping children:

“I am agreeing to it because I think it might help kids… I am hoping it helps other children as well.” (MB Katie)

### Personal benefits

A key motivator for taking part in the research was one of self-interest. Three sub-themes were identified: (1) participating to aid the return of their child/ren from care via the perceived mechanism of co-operation; (2) participating in order to gain access to services and support; and (3) participating to help their future selves.

### Participating to aid the return of their child/ren from care via the perceived mechanism of co-operation

For participants whose children were recently taken into care (predominately MB Mothers), participating in research was about being seen to cooperate with social work processes and increase their likelihood of having their child/ren returned. Although the research is separate to social work services, participation was seen as part of a bigger picture of trying to get their children back, inextricable from other proceedings:

“It is just part of getting my girls home… I thought it would help on my part as well, like to help to get the kids back.” (MB Eleanor)

This aim to be seen as co-operative above all else meant that the position and reality of voluntariness was sometimes questioned. In the context of desperation for the speedy return of their child/ren, the notion of having choice over participation was seen as illusive even although understood by mothers as an explicit aim of the research process:

“[There was] a choice not to sign up, but no choice because we were trying to look for a parenting assessment so at that time it was sort of a good time to sign up because it was the last option. I just sort of signed up and then got information later.” (MB Paula)

“It’s happening whether you like it or not.” (MB Alice)

In seeking to demonstrate co-operation, mothers in the MB group sometimes expressed a sense of confusion about the distinction between the research and social care proceedings despite clarification from the research team. This led some to perceive that their participation may be encouraged or endorsed by social work services. In the absence of certainty on this point, mothers were keen to participate *in case* it was seen as favourable by social work services. In other words, they were prepared to ‘cover all bases’ when it came to demonstrating their willingness to cooperate in the hope of expediting the return of their child/ren:

“I wasn’t really clear if this was actually something social work wanted me to do or if it was just completely voluntary on my behalf.” (MB Janice)

However, this can be contrasted with the mothers in the MR group which included some mothers who were still undergoing assessment. Mothers in this study did not consider participation as a potential means of reunification possibly indicating that the setting and context of the research—recruited via social services prior to their parenting capacity assessment versus through their Alcohol and Drug Recovery Service—had an impact on both their understanding of the studies and their motivations for participation.

### Participating to gain access to services and support

Some mothers perceived participation as a chance to address the issues that led to them having their child removed, with the MB mothers having the aim of improving their parenting:

“I was willing to do anything or try and kind of grasp onto anything I could do towards like parenting classes or support…anything I could do regarding work on myself to get my daughter back.” (MB Janice)

This sub-theme also housed perceptions about a need for support more broadly than the return of children from care from both groups of mothers:

“I’ve got loads of demons to work through.” (MR Annie)

High levels of unmet need in relation to parenting and their own mental health were apparent in the interviews:

“It’s very, very frustrating… My son is coming up for a year and I’m still waiting for my therapy and everything else.” (MB Amber)

It is possible women were using the interviews to meet with professionals to discuss this unmet need and potentially access alternative sources of support that were not provided through their current support systems. Annie (MR), for example, asked if she could attend the Women’s Trauma Group run by LR: “I can still work with you and [Nurse co-facilitator] and do all that, can’t I?”

Mothers (predominately the MR group) also highlighted that they felt that contact with child welfare services and the removal of children was a difficult experience which exacerbated their pre-existing issues and increased their need for further support:

“That’s a huge trauma, losing your child.” (MR Lisa)

### Participating to help their future selves

Although mothers had insight into their current circumstances, most of the mothers, and particularly the MR group, also had hope that, one day in the future, they might be able to take their knowledge and experience and work with other mothers who have had children removed:

“I am not fit to do work or anything, but if I did that’s what I would do, addiction or…and to help mothers out there.” (MR Lynne)

Mothers, again, positioned this aspiration within the context of a lack of support:

“I would love to work, you know once I have dealt with all this stuff, in the future I would love to be able to work with parents of accommodated children because there is a total feeling of abandonment.” (MR Toni)

While not able to work or provide the support they would like to at this time, by participating in research the mothers were able to perceive this as a step towards this goal and a means of being able to work and support mothers in a wider sense at a time where they were limited in their own ability to do so.

### Empowerment

The data highlighted feelings of disempowerment experienced by mothers with addiction issues involved in the child welfare system, with taking part in research being viewed as a chance to speak up and to make change. This theme has two sub themes: (1) being heard and (2) giving voice to others.

### Being heard

Most of the mothers felt that the social work processes they were involved in were focussed on their children:

“The whole process from start to finish, it was all about my child which is understandable.” (MR Lisa)

But this came at the risk of not having their own stories and experiences fully understood. Mothers, particularly those with more time between assessment/removal and the interview, spoke about wanting to have their own voices heard. Participating in the research provided them with an opportunity to discuss their experience from their point of view and to tell their own story or version of events. This was felt as empowering:

“This is the first time I’ve ever been asked anything about how it feels to lose my children.” (MR Annie)

“The main thing is being heard.” (MR Shona)

Some felt the information about themselves in social work reports was out of date or incorrect; by agreeing to the interview, they had an opportunity to tell their own story, with no possibility of an alternative version being construed.

Beyond being heard and “setting the record straight,” mothers recognised the impact that the lack of voices of mothers may have on research or service development and planning. Researchers listening first-hand to their stories and experiences was seen as vital in developing and improving services:

“Obviously you [researcher] are doing a lot of reading, but you are also coming to see people, you are getting involved and you are doing it right.” (MR Annie)

Mothers felt that there was a whole narrative behind actions and decisions that needed unearthed in the research process. This was seen as integral to providing context to situations where children are removed:

“I felt beforehand this could be really worthwhile, you know, a study taking place for, like, others to look from outside the box to see what is going on here, and yeah I was really quite, you know, I wanted to do it and really grasped onto it.” (MB Janice)

“We do not just have kids and give them away, that’s not the way it is.” (MR Jess)

Participating in research also gave mothers the opportunity to provide advice and feedback to social work services. A need for a more compassionate and constructive approach was highlighted, which mothers felt would aid the ability to change via focusing on *how* to improve. The existing approach was often seen as deficit-focussed:

“I know they have got to do their looking into things and making sure things are as they are supposed to be, but constantly getting told that you were untidy, unclean or did not have enough food, there was never any …this is what you need to do…or anything like steering you in the right direction.” (MR Shona)

### Giving voice to others

During their interviews, mothers talked about other women they knew who had their children removed or more generally about the population of women who have had children removed.

Mothers who had support from partners, family, or friends or who had additional resources (such as finances, knowledge of the system or a lawyer) reported concerns about women without these same opportunities. They described feeling worried about these women and wanted to participate to increase knowledge and support for mothers who have had children removed but who may not have the same ability as themselves to be heard. Although related to notions of altruism, this sub-theme illuminates a more specific wish to “help” that represents a desire of mothers to not only be given their own voice, but to also be a voice and advocate for other mothers. This particular group of mothers saw participating in research as a mechanism by which inequalities in mothers’ abilities to be heard could be redressed by those able to use their voice, representing more than just their own views:

“You are just kind of left to flounder on your own and I am fortunate that I was able to source and get and ask and do, but it is for the people who…have come from the council schemes [social housing in areas of socioeconomic deprivation], the broken homes, the non-educated or whatever… haven’t got the ability.” (MR Toni)

“If it wasn’t for my mother, I do not know how I would have probably dealt with all that myself, probably would not have, and that’s a shame because the thought of all those mothers that do not have that support, and just get their weans [children] taken away and that’s it, and then they give up.” (MR Lynne)

## Discussion

Mothers gave rich descriptions of their reasons for taking part in research. The themes suggest that participation in research for this group is multi-faceted, just like other participant groups (24–26). While aiming to improve their own situation was a key motivator for participation, mothers also gave descriptions of participation that included benefits related to empowerment, an ability to help and to ‘finally have a voice’. Placing these themes in the context of their wider life circumstances, participants conveyed an overarching sense that they have limited opportunity elsewhere to have these needs met. In essence, mothers participated on the perception that this would help their current and future circumstances but also to help others.

Similar to previous research, our results show it is possible for vulnerable groups to participate in research at challenging times and to discuss sensitive topics. Mothers wanted to help and could make decisions about potential benefits to themselves and others if they participate. Our results add support to the concept that altruism and social change are powerful motivators for participation (24, 27); in addition to personal benefits (28, 29) including being able to access treatment or improve quality of life (30).

Previous research has highlighted that motivations vary between and within groups. For example, financial rewards are motivating but not the most common reason for participating (30, 31) and can be more likely to motivate certain groups such as younger participants or those with financial pressures (30). Our results expand on this by showing that the passage of time has an impact on motivation. While there were common motivations across both groups, only mothers currently undergoing assessment were motivated by a perception that co-operation may help with reunification with their children. On the other hand, mothers who had completed their assessments and had time to reflect on the process were more likely to be motivated by the thought of helping their future selves and using their voice for advocacy for other mothers and to shape/influence services.

The data comes from two different studies, and we believe that combining and synthesising specific elements of the original datasets was justified and strengthened the analysis and conclusions. By having each stage of the analysis conducted by a different author and then reviewed by an expert by experience, we hope to have reduced any possible bias. However, despite combining data from two different studies, the participants were all recruited from the same area in Scotland, increasing the likelihood of homogenous experiences and responses may have differed if the mothers were recruited from other areas. As this study used secondary data from two different studies, there are limitations with this approach as the studies had separate aims. The data about participation was taken from the wider interviews and not all questions were related to this area. All the mothers discussed their motivations and reasons for participation in their interviews, some without prompting, highlighting this as an important issue for them. However, we may have obtained more detailed responses if asking more directly about participation or if the interviews focused more in this area. As this study only interviewed those who participated in research, we lack the views of those who chose not to participate. Similarly, while we focused on why the mothers participated, it would be beneficial to explore barriers to participation and strategies to increase participation as this information could shape future research with this group.

### Final reflections by expert by experience

SG—“Reflecting on this paper reminds me of the many times where I, and lots of women I have worked with, have felt unheard and just wanted someone to listen to how our lives could be made better had we been asked what we needed as there can be a real lack of understanding. As I read over this paper, I remember someone asking me research questions even though I was seen as vulnerable, and it changed my life for the better in so many ways. It’s the reason I believe that research is hugely beneficial and greatly needed so that a way can be found to make the lives of women in addiction a better one, which will then in turn give children a better chance at life too. I think this paper shows that.”

## Conclusion

This study has shown that despite being ‘doubly vulnerable’ these mothers wanted to participate in research for their own benefit and the benefit of others. However, research needs to remain mindful of issues about consent and vulnerability and designing inclusion, exclusion and referral pathways appropriately, and with the right processes and safeguarding in place (14), as we have a responsibility to conduct research that is relevant to all members of the population. When a population is ‘doubly vulnerable’, it is even more crucial that this research is done. It is only the ‘doubly vulnerable’ who can answer certain research questions (13); otherwise, appropriate interventions cannot be developed to improve treatment options and services and to reduce drug-related deaths and the impact that addiction has on women and their children. We need to listen to these voices.

## Data availability statement

The datasets presented in this article are not readily available as ethical approval and participant consent was only provided for quotes from the datasets and was not proivde to share the full datasets. Requests to access the datasets should be directed to the corresponding author.

## Ethics statement

The studies involving human participants were reviewed and approved by West of Scotland NHS REC. The patients/participants provided their written informed consent to participate in this study. Written informed consent was obtained from the individuals for the publication of any potentially identifiable images or data included in this article.

## Author contributions

LR, KC, and FT contributed to the conception, design, and data analysis. LR and KC wrote the first draft of the manuscript. SG wrote a section of the paper and provided reflections as an expert by experience. All authors contributed to the article and approved the submitted version.
