# Supplementary material for: Helping themselves and helping others: how the passage of time influences why mothers with addictions take part in research
Source: Front Psychiatry. 2023 Oct 4;14:1204882. doi: 10.3389/fpsyt.2023.1204882 (PMC10582753; doi:10.3389/fpsyt.2023.1204882)
Supplement: Supplementary file 1 [file Data_Sheet_1.docx]

Supplementary Material

Study aims:

Study 1:

Study 1 is a nested qualitative study contributing to the process evaluation of an RCT.  The nested study explores issues of consent and participation.

Study 2:

A qualitative (IPA) study to explore mothers’ experiences of child removal and support from services in the context of addiction.

Inclusion/Exclusion Criteria

Study 1:

Inclusion

- Birth mother who had consented to the main RCT and
- Had agreed to be contacted about an additional qualitative interview

Exclusion

- Unable to speak English
- Were excluded or unable to participate in the RCT intervention
- Concerns raised by social worker about ability to consent or engage in interview or concerns that participation may exacerbate current difficulties (such as sudden deterioration in mental health, recent bereavement, recent adverse reaction to permanence decision)

Study 2:

Inclusion

- Women who are over 18 years
- Fluent in English
- Current service users of Drug and Alcohol Recovery Service
- Have a child or children removed from their care by social work

Exclusion

- Women who are under 18 years of age
- Unable to speak English
- Unable to consent due to being under the influence of alcohol and/or substances
- Do not have children in their care but no social work involvement i.e. they live with their father or another family member informally rather than in a kinship arrangement
- Current or previous patient of Chief Investigator

Current Study

Inclusion

- Women who participated in Study 1 or 2
- Current or history of Addiction issues

Exclusion

- No addiction issues

Interview guides

Study 1:

Mothers were asked about their experiences of being recruited to the trial, why they decided to take part, their thoughts on trial processes, their experiences of the parenting capacity assessment they received and what impact this had on them and their parenting, where relevant.

Study 2:

Mothers were asked about their experience of having their children removed, what services they were working with at the time of the removal and any that were offered during or after removal, their experience of contact with services, what they felt mothers needed from services at different stages of the removal process and their advice for services who work with these women.
